# Laser-Assisted Subepithelial Keratectomy versus Laser In Situ Keratomileusis in Myopia: A Systematic Review and Meta-Analysis

**DOI:** 10.1155/2014/672146

**Published:** 2014-06-12

**Authors:** Li-Quan Zhao, Huang Zhu, Liang-Mao Li

**Affiliations:** ^1^Department of Ophthalmology, No. 181 Hospital of the PLA, Guilin, Guangxi 541002, China; ^2^Department of Ophthalmology, Xinhua Hospital Affiliated to Shanghai Jiao Tong University School of Medicine, Shanghai 200092, China

## Abstract

This systematic review was to compare the clinical outcomes between laser-assisted subepithelial keratectomy (LASEK) and laser in situ keratomileusis (LASIK) for myopia. Primary parameters included mean manifest refraction spherical equivalent (MRSE), MRSE within ±0.50 diopters, uncorrected visual acuity (UCVA) ≥20/20, and loss of ≥1 line of best-corrected visual acuity (BCVA). Secondary parameters included flap complications and corneal haze. Twelve clinical controlled trials were identified and used for comparing LASEK (780 eyes) to LASIK (915 eyes). There were no significant differences in visual and refractive outcomes between the two surgeries for low to moderate myopia. The incidence of loss of ≥1 line of BCVA was significantly higher in moderate to high myopia treated by LASEK than LASIK in the mid-term and long-term followup. The efficacy (MRSE and UCVA) of LASEK appeared to be a significant worsening trend in the long-term followup. Corneal haze was more severe in moderate to high myopia treated by LASEK than LASIK in the mid-term and long-term followup. The flap-related complications still occurred in LASIK, but the incidence was not significantly higher than that in LASEK. LASEK and LASIK were safe and effective for low to moderate myopia. The advantage of LASEK was the absence of flap-related complications, and such procedure complication may occur in LASIK and affect the visual results. The increased incidence of stromal haze and regression in LASEK significantly affected the visual and refractive results for high myopia.

## 1. Introduction

Since laser-assisted subepithelial keratectomy (LASEK) was introduced to refractive surgery in recent decade, many clinical controlled trials have been reported [1–12]. Although laser in situ keratomileusis (LASIK) is dominantly used in refractive surgery due to little pain and rapid visual rehabilitation, surface ablation including LASEK is also a valuable technique for high myopia, thinner cornea, or retinal pathology [13, 14]. Some studies reported that LASIK seemed to have significant benefit over LASEK [12]. There are also conflicting reports about the postoperative visual recovery between the two surgeries [2].

Therefore, it is necessary to review in greater depth the available studies to understand the benefits of LASEK versus LASIK in myopic patients. Due to a scarcity of randomized control trials (RCTs) addressing this issue we decided to include nonrandomized comparative studies. We performed a meta-analysis of existing RCTs and comparative studies of LASEK versus LASIK for the treatment of myopia in an attempt to detect any differences in safety and efficacy as the primary concern between the two techniques. Corneal haze and flap-related complications influence final visual outcomes. These 2 key variables were chosen as secondary outcome parameters in this study.

## 2. Materials and Methods

### 2.1. Literature Search

Two reviewers independently searched the following electronic databases: PubMed, EMBASE, and the Cochrane Controlled Trials Register up to July 6, 2012. To achieve the maximum sensitivity of the search strategy and identify all trials comparing LASEK and LASIK, we used appropriate free text and thesaurus terms including “Laser-Assisted Subepithelial Keratectomy” or “Laser Subepithelial Keratomileusis” or “Laser-Assisted Subepithelial Keratomileusis” or “Subepithelial Photorefractive Keratectomy” or “LASEK” and “Laser In Situ Keratomileusis” or “LASIK.” After the relevant titles were identified, the abstracts and full text of these studies were reviewed to decide whether they met criteria for our study. A manual cross-reference search of the bibliographies of relevant articles was conducted to identify studies not found through the computerized search. The “related articles” feature of PubMed was used as well.

The search included all controlled clinical trials and comparative studies comparing LASEK and LASIK for myopia. Patients were presented with any degree of myopia and astigmatism and were aged greater than 18 years. At least one or more clinical outcome parameters representing visual acuity, spherical equivalent, corneal haze, or flap complications must be assessed and published. In the selected studies, standard surgical techniques were required: alcohol application to remove corneal epithelium in LASEK. Mitomycin-C (MMC) was not used in any of the studies. There was no language restriction on the publications.

### 2.2. Quality Assessment

The study quality assessment was based on the methods recommended in the* Cochrane Handbook for Systematic Reviews of Interventions* [15]. Areas of methodological quality examined were randomization, allocation concealment, masking of outcome assessment, and completeness of follow-up. According to potential bias from included studies, we selected the following: one patient treated with two surgeries (?), similar preoperative manifest refraction spherical equivalent (MRSE) (?), similar best-corrected visual acuity (BCVA) (?), similar pachymetry (?), one surgeon (?), one machine (?), and timing of the outcome assessment in two similar groups (?) as quality assessment index. Based on the Cochrane Collaboration guidelines and these added assessments, the selected studies were appraised by two reviewers independently. The results were compared and found to be identical.

### 2.3. Data Extraction

Data were extracted by the two reviewers independently on a preformatted sheet. The primary outcome parameters for inclusion were safety and efficacy on the basis of the previous study [16]. Efficacy measures examined were proportions of patients achieving uncorrected visual acuity (UCVA) ≥20/20 and MRSE within ±0.50 diopters (D) of the target. Safety measures examined were proportions of patients losing ≥1 line of BCVA and final mean MRSE. The proportion of flap-related complications and corneal haze (higher than Grade 1) were assessed as the secondary outcome parameters. A customized data extraction form, as described in the* Cochrane Handbook for Systematic Reviews of Interventions*, was used to record the authors of each study, the year of the trial, duration of the study, the number of subjects, demographics information of each study subject, and the laser machine type used in the procedures. The preoperative mean MRSE and mean BCVA were also recorded. According to diopters, the included studies were divided into two groups: low to moderate myopia group (less than −4.50 D) and moderate to high myopia group (higher than −4.50 D).

### 2.4. Statistical Analysis

Quantitative data for the minimal outcome criteria were entered into the software Cochrane Review Manager (RevMan) version 5.0 and analyzed. Summary estimates, including 95% confidence intervals (CI), were calculated. For continuous outcome data (e.g., mean MRSE) means and standard deviations were used to calculate a weighted mean difference (WMD). For dichotomous outcomes (e.g., proportions of UCVA ≥20/20), the odds ratio (OR) was calculated.

Statistical heterogeneity was tested using *χ* ^2^ and *I* ^2^ tests. Fixed effects model was used unless significant evidence of statistical heterogeneity or clinical diversity was found. However, for results showing significant heterogeneity (*I* ^2^ > 50%), random-effects meta-analysis was performed. Outcome measures were assessed on an intent-to-treat (ITT) basis. A *P* value less than 0.05 was considered statistically significant.

Publication bias was assessed by visually inspecting a funnel plot.

## 3. Results

The combined search identified a total of 23 publications. Eleven studies were excluded after abstract evaluation. Twelve studies published between 2002 and 2008 met the inclusion criteria [1–12]. In the end, 1 study [2] in RCT and 11 nonrandomized comparative studies [1, 3–12] were included in the present meta-analysis involving a total of 1011 patients who underwent LASEK or LASIK. The selection of 12 studies is summarized in Table 1. We identified several potential sources of bias in the included studies (Table 2).

### 3.1. Outcomes of Low to Moderate Myopia

**Figure 1:**
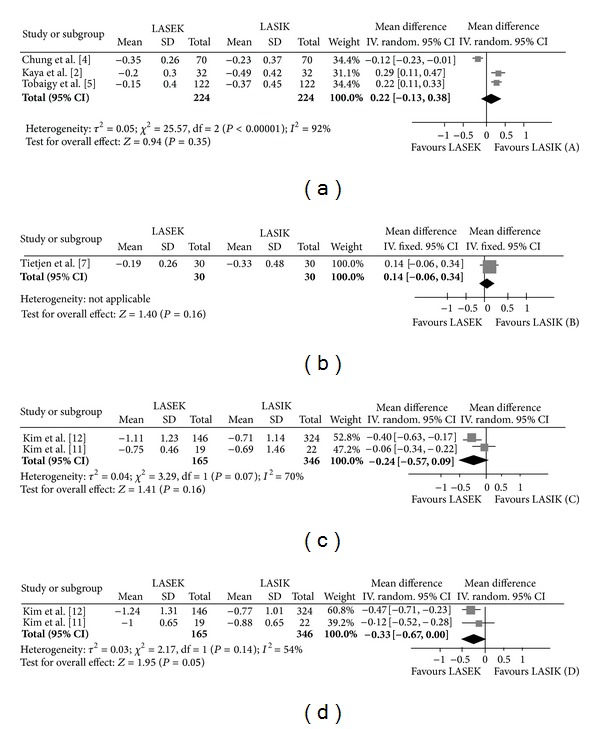
Forest plots of mean difference of mean refractive spherical equivalent comparing LASEK to LASIK for low to moderate myopia at 6 months (a) and 12 months (b) postoperatively and for moderate to high myopia at 6 months (c) and 12 months (d) postoperatively. LASEK: laser-assisted subepithelial keratectomy. LASIK: laser in situ keratomileusis.

Six months postoperatively, there were no statistically significant differences in mean MRSE, the proportion of the refractive SE within ±0.50 D, and the proportion of loss of ≥1 line of BCVA between the LASEK and LASIK groups (*P* = 0.35, *P* = 0.10, and *P* = 0.33, resp.) (Figure 1(a) and Table 3). The severity of corneal haze was also not significantly different between the two groups (Table 3).

Twelve months postoperatively, there were no statistically significant differences in mean MRSE, the proportion of the refractive SE within ±0.50 D, UCVA ≥20/20, and loss of ≥1 line of BCVA between the LASEK and LASIK groups (*P* = 0.16, *P* = 0.69, *P* = 0.39, and *P* = 1.00, resp.) (Figure 1(b) and Table 3).

### 3.2. Outcomes of Moderate to High Myopia

Six months postoperatively, there were no statistically significant differences in mean MRSE, the proportion of the refractive SE within ±0.50 D, and the proportion of UCVA ≥20/20 between the LASEK and LASIK groups (*P* = 0.16, *P* = 0.85, and *P* = 0.19, resp.) (Figure 1(c) and Table 3). The proportion of participants with loss of ≥1 line of BCVA in the LASEK group was significantly higher than that in the LASIK group (*P* = 0.0004) (Table 3). Corneal haze was more severe in the eyes treated with LASEK than those treated with LASIK (*P* < 0.00001) (Table 3).

Twelve months postoperatively, there were no statistically significant differences in the proportion of the refractive SE within ±0.50 D between the two groups (*P* = 0.85) (Table 3). There was an increasing trend of statistically significant difference in mean MRSE for the LASEK-treated eyes (*P* = 0.05) (Figure 1(d)). The proportion of UCVA ≥20/20 was significantly less (*P* = 0.02), and the proportion of loss of ≥1 line of BCVA was significantly more (*P* < 0.00001) in the LASEK-treated eyes than the LASIK-treated eyes (Table 3). Corneal haze was more severe in the eyes treated with LASEK than those eyes treated with LASIK (*P* < 0.00001) (Table 3).

### 3.3. Flap Complications

Three studies reported flap complications [1, 8, 12]. Two studies indicated that no intraoperative or postoperative flap complications occurred in the LASIK-treated eyes [1, 8]. The remaining study reported that corneal flap displacements were found on postoperative day 1 in 4 LASIK-treated eyes [12]. Analysis of these data showed no significant difference in the incidence rate of flap-related complications between two groups (*P* = 0.34) (Table 2).

**Figure 2:**
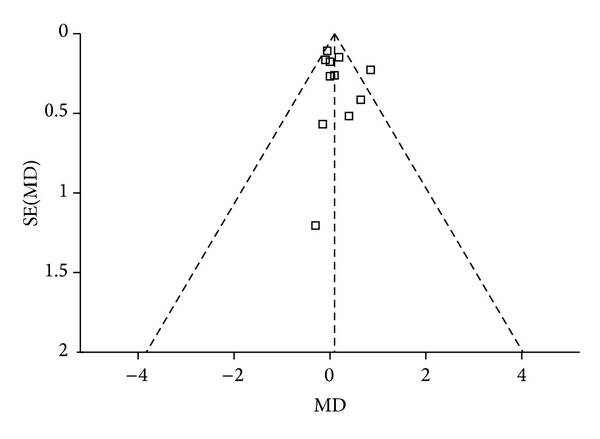
Funnel plot of clinical controlled trials included in the meta-analysis.

Based on a visual analysis of the funnel plots, no obvious evidence of publication bias was founded (Figure 2).

## 4. Discussion

With the evidence available from the selected clinical controlled trials, we found that there were no significant differences in visual and refractive outcomes between the LASEK and LASIK for low to moderate myopia in the midterm and long-term follow-up. However, the safety profile (loss of ≥1 line of BCVA) of the LASEK-treated eyes in moderate to high myopia was significantly worse than that of the LASIK-treated eyes in the midterm and long-term follow-up. The efficacy (MRSE and UCVA) of LASEK for moderate to high myopia appeared to be a significantly worsening trend than those of LASIK in the long-term follow-up.

Corneal haze is a known risk factor and has been extensively investigated. There was no significant corneal haze (higher than Grade 1) in LASEK-treated eyes at 3 and 6 months postoperatively due to low to moderate myopia [1, 2, 8, 9]. However, corneal haze occurred more frequently (25.3%) at 6 and 12 months postoperatively in Kim et al.'s study [12] due to high myopia, conforming to the results of a previous study by Lin et al. [17]. The increased usage of surface ablation techniques for high myopia is ascribed to cause the higher incidence of clinically significant haze and regression in LASEK.

The visual and refractive results (UCVA, BCVA, and MRSE) at 6 or 12 months postoperatively indicated no statistically significant differences between the two groups in subjects with low to moderate myopia [2, 5]. Additionally, the normal visual function, such as reading performance after refractive surgery, was not significantly changed in the two groups [6]. Even in de Benito-Llopis et al.'s study, the Snellen BCVA levels and safety index (postoperative BCVA/preoperative BCVA) were significantly higher in the LASEK group than those in the LASIK group [1].

In other more sensitive parameters, such as contrast sensitivity (CS) and higher-order aberrations (HOAs), Kim's study and Tietjen et al.'s study all demonstrated that CS values of the LASEK group were higher than those in the LASIK group at 6 and 12 months postoperatively [7, 9]. In Kirwan and O'keefe's study, a greater increase in HOAs was induced by LASIK rather than that by LASEK treatments [3]. These differences in objective parameters may support the belief that better visual quality can be achieved by LASEK in low to moderate myopia. Some authors thought that flap and microkeratome complications or interface problems (cutting of the LASIK flap and subsequent repositioning onto the stromal bed) seemed to be the main factors responsible for the postoperative increasing in HOAs reported in Kirwan and O'keefe's study [3]. Other authors believed that the reduction of CS was caused by stromal wound healing-related factors after creation of the flap, not by the deeper ablation depth [2, 7].

Spherical-like aberrations were significantly greater after LASEK than those after LASIK at postoperative 3 months in Buzzonetti et al.'s study [10]. Authors thought that the difference may be due to the central subepithelial fibroblast hyperplasia (we thought it was corneal haze) observed after LASEK. Compared to Kirwan and O'keefe's study, we thought that the duration of follow-ups was short and the degree of myopia was higher in Buzzonetti et al.'s study, which resulted in differences in aberrations. The differences of visual results conformed to the different incidence of corneal haze between the two different degrees of myopia in the present meta-analysis.

Three studies reported flap-related complications [1, 8, 12]. The incidence rate of flap-related complications of LASIK was not significantly higher than that of LASEK in the present meta-analysis. However, reduced BCVA in LASIK-treated eyes is known to be associated with flap-related complications including an inconspicuous shifted flap, striae, and interface debris. Refractive surgeons need to be aware of the potential risk of postoperative flap-related complications such as diffuse lamellar keratitis, epithelial ingrowth, flap infection, or flap traumatic avulsion [18]. And we must take caution to identify these complications in the early and late postoperative periods and provide effective management.

To summarize, the present meta-analysis study of LASEK versus LASIK seemed to suggest that both procedures were safe, effective, stable, and predictable for the treatment of low to moderate myopia. Advantages of LASEK may include the absence of stromal wound healing-related factors or flap-related complications that can occur in LASIK, which may decrease in BCVA and contrast sensitivity or increase higher-order aberrations. However, the increased incidence of stromal haze and regression in LASEK significantly affected the visual and refractive results in high myopia. Recently, studies showed that the intraoperative MMC in LASEK or PRK could reduce corneal haze and regression and result in similar visual and refractive outcomes compared to LASIK for moderate to high myopia [19, 20]. Further evidence-based study will be needed to clarify the advantages of LASEK/PRK with intraoperative MMC compared to LASIK.

Some limitations of the present meta-analysis should be acknowledged.

First, in our selected 12 studies, 1 study is RCT and the remaining 11 studies are retrospective comparative studies or prospective nonrandomized controlled trials. Theoretically, the principle of meta-analysis is to collect data from prospective randomized controlled trials and perform valid comparison [21, 22]. The low number of RCTs reported in the literature may be ascribed to the different procedural criteria applied in clinical practice for LASEK and LASIK due to severity of myopia and/or thin cornea. It is impossible to perform randomization, allocation concealed, or double-blinded during comparative study due to the surgical difference. In all of the selected studies, allocation to the two groups was not reported. Even in the only RCT, the randomization process was not described [2]. This may introduce selection bias in allocating interventions to participants.

Second, there were many potential biases between the groups in each study according to Table 3. There were differences in preoperative patient characteristics such as MRSE, BCVA, and pachymetry [3, 5, 9, 11]. It is recognized that surgical nomograms, laser systems, surgical experiences, and intra- and postoperative procedures affect the clinical outcomes. Surgeon, machine, and timing of the outcome assessment in the groups (follow-up intervals) had obvious differences in the selected studies. Therefore, the results should be interpreted with caution due to this heterogeneity. Access to individual level data could certainly have improved the quality of adjustment as well as the precision of estimates.

Third, the group allocation according to diopters was not exactly accurate. Some included studies did not report the range of myopia in detail. We roughly calculated 80% confidence intervals of diopters according to mean preoperative MRSE and standard deviations. There were overlapping parts between the low to moderate myopia group and the moderate to high myopia group.

Finally, we cannot fully exclude publication bias. There were no sufficient studies to detect asymmetry in a funnel plot. In addition, we did not attempt to gain access to unpublished results, and only studies written in English were selected.

## Figures and Tables

**Table 1:** Characteristics of studies of LASEK versus LASIK included in the meta-analysis.

Trial (location)	Laser machine	Duration (m)	Eyes	Men (%)	Age (years) Mean ± SD (range)	MRSE (D) Mean ± SD (range)	BCVA
Low to moderate							

de Benito-Llopis et al. 2007 (Spain) [1]	Technolas 217C	3	LASEK: 79 LASIK: 79	NA NA	31.6 ± 6 (19–46) 33.3 ± 8 (20–66)	−1.59 ± 0.80 −1.53 ± 0.60	1.20 ± 0.1 1.17 ± 0.1

Kaya et al. 2004 (Turkey) [2]	LaserSight LSX	6	LASEK: 32 LASIK: 32	56.3 56.3	total: 26.83 ± 5.33 (19–36)	−2.69 ± 1.65 −3.09 ± 2.44	1.00 ± 0.06 0.97 ± 0.10

Kirwan and O'Keefe 2009 (Ireland) [3]	Technolas 217z laser	12	LASEK: 50 LASIK: 65	NA NA	32.2 ± 9.2 (20–52) 33.5 ± 6.7 (22–51)	−3.3 ± 1.6 (−1.1 to −8.6) −3.4 ± 1.1 (−1.5 to −6.5)	100% ≥ 6/6

Chung et al. 2006 (Korea) [4]	VISX Star S4	6	LASEK: 70 LASIK: 70	NA NA	NA	−3.33 ± 0.57 −3.52 ± 1.13	1.00 ± 0.03 1.00 ± 0.02

Tobaigy et al. 2006 (USA) [5]	Technolas 217z VISX Star S2, S4	6	LASEK: 122 LASIK: 122	58.5 51.9	34.77 ± 7.49 (21–53) 34.95 ± 7.73 (21–61)	−3.50 ± 1.40 −3.50 ± 1.42	NS

Richter-Mueksch et al. 2005 (Austria) [6]	Alcon LADARVision Allegretto Wave excimer laser	3 wks	LASEK: 26 LASIK: 26	52.9 47.1	33.9 ± 6.9 (21–38) 34.8 ± 6.2 (20–37)	−4.00 ± 2.1 (−1.0 to −9.5) −3.58 ± 2.0 (−1.0 to −8.5)	NS

Tietjen et al. 2008 (German) [7]	MEL 70G Excimer laser	12	LASEK: 30 LASIK: 30	50.0 50.0	total: 35 (18–52)	−3.90 ± 1.57 −4.54 ± 1.64	0.89 0.94

Teus et al. 2007 (Spain) [8]	Technolas 217C	3	LASEK: 40 LASIK: 40	NA NA	31.0 ± 6.0 (20–42) 32.6 ± 4.8 (21–39)	−4.20 ± 1.20 (−2.00 to −6.40) −4.20 ± 1.20 (−2.00 to −6.40)	NA

Moderate to high							

Kim et al. 2007 (Korea) [9]	VISX Star S4	6	LASEK: 148 LASIK: 87	33.0 33.3	27.05 ± 6.56 27.33 ± 5.63	−4.54 ± 1.72 (−1.0 to −8.75) −5.39 ± 1.70 (−1.5 to −11.5)*	−0.07 ± 0.08 −0.06 ± 0.11

Buzzonetti et al. 2004 (Italy) [10]	Alcon LADAR-Vision 4000	3	LASEK: 18 LASIK: 18	NA NA	33.6 ± 8.8 38.8 ± 11.08	−5.70 ± 4.05 −5.4 ± 3.1	NA

Kim et al. 2005 (Korea) [11]	VISX Star	24	LASEK: 19 LASIK: 22	30.0 28.6	25 (23–29) 25 (24–27)	−8.50 (−6.81 to −9.69) −7.49 (−7.25 to −9.69)	NA

Kim et al. 2004 (Korea) [12]	Nidek EC-5000	12	LASEK: 146 LASIK: 324	27.4 32.9	27.91 ± 4.31 (20–41) 28.37 ± 6.71 (21–39)	−8.01 ± 1.85 (−6.00 to −12.50) −7.91 ± 1.26 (−6.00 to −11.50)	NA

LASEK = laser-assisted subepithelial keratectomy, LASIK = laser in situ keratomileusis, MRSE = manifest refraction spherical equivalent, D = diopter, BCVA = best-corrected visual acuity, m = month, wks = weeks, NS = not significant, NA = not available.

**Table 2:** Quality appraisal of included studies.

	de Benito-Llopis et al. [1]	Kaya et al. [2]	Kirwan and O'Keefe [3]	Chung et al. [4]	Tobaigy et al. [5]	Richter-Mueksch et al. [6]	Tietjen et al. [7]	Teus et al. [8]	Kim et al. [9]	Buzzonetti et al. [10]	Kim et al. [11]	Kim et al. [12]
Prospective (?)	Yes	Yes	Yes	Yes	No	Yes	Yes	No	No	Yes	No	Yes
Randomization (?)	No	Yes	No	No	No	No	No	No	No	No	No	No
Concealed allocation (?)	No	No	No	No	No	No	No	No	No	No	No	No
Blinded outcome assessors (?)	Yes	No	No	No	No	No	No	Yes	No	No	No	No
Completeness of follow-up (?)	Yes	Yes	Yes	Yes	Yes	Yes	Yes	Yes	Yes	Yes	Yes	Yes
One patient treated with two surgeries (?)	No	Yes	No	No	No	No	Yes	No	No	No	No	No
Similar MRSE (?)	Yes	Yes	Yes	Yes	Yes	Yes	Yes	Yes	No	Yes	Yes	Yes
Similar BCVA (?)	Yes	Yes	Yes	Yes	Yes	Yes	Yes	Unclear	Yes	Unclear	Unclear	Unclear
Similar pachymetry (?)	Unclear	Yes	No	Unclear	No	Unclear	Yes	Yes	Unclear	Unclear	No	Unclear
One surgeon (?)	Yes	Unclear	Yes	Unclear	Yes	Unclear	Yes	Yes	Yes	Unclear	Yes	Yes
One machine (?)	Yes	Yes	Yes	Yes	Yes	Yes	Yes	Yes	Yes	Yes	Yes	Yes
Timing of the outcome assessment similar (?)	Yes	Yes	Yes	Yes	No	Yes	Yes	Yes	Yes	Yes	Yes	Yes

**Table 3:** Postoperative course and complications of LASIK versus LASEK in the meta-analysis.

	Number of studies	Crude rate, *n/N* (%)		Rate difference % (95% CI)	*P* for overall effect
		LASEK	LASIK		
Low to moderate					
After 6 months					
Refractive SE ≤ 0.5 D	1 [5]	98/122	87/122	1.64 (0.91, 2.98)	0.10
UCVA ≥ 20/20	NA	—	—	—	—
Loss of ≥1 line of BCVA	2 [2, 5]	7/154	11/154	0.61 (0.23, 1.64)	0.33
Corneal haze	1 [2]	0/32	0/32	Not estimated	Not estimated
After 12 months					
Refractive SE ≤ 0.5 D	2 [3, 7]	77/80	91/95	1.38 (0.28, 6.80)	0.69
UCVA ≥ 20/20	1 [7]	23/30	20/30	1.64 (0.53, 5.12)	0.39
Loss of ≥1 line of BCVA	2 [3, 7]	5/95	5/95	1.00 (0.26, 3.89)	1.00
Corneal haze	NA	—	—	—	—
Moderate to high					
After 6 months					
Refractive SE ≤ 0.5 D	1 [12]	101/146	227/324	0.96 (0.63, 1.47)	0.85
UCVA ≥ 20/20	1 [12]	92/146	224/324	0.76 (0.50, 1.15)	0.19
Loss of ≥1 line of BCVA	1 [12]	16/146	8/324	4.86 (2.03, 11.64)	0.0004
Corneal haze	2 [9, 12]	37/294	2/411	54.65 (12.96–230.52)	<0.00001
After 12 months					
Refractive SE ≤ 0.5 D	1 [12]	101/146	227/324	0.96 (0.63, 1.47)	0.85
UCVA ≥ 20/20	1 [12]	88/146	232/324	0.60 (0.40, 0.91)	0.02
Loss of ≥1 line of BCVA	1 [12]	25/146	4/324	16.53 (5.64, 48.48)	<0.00001
Corneal haze	1 [12]	37/146	2/324	54.65 (12.96–230.52)	<0.00001
Flap complications	3 [1, 8, 12]	4/443	0/265	0.24 (0.01–4.54)	0.34

LASIK: laser in situ keratomileusis, LASEK: laser-assisted subepithelial keratectomy, D: diopter, SE: spherical equivalent, UCVA: uncorrected visual acuity, BCVA: best-corrected visual acuity, and NA: not available.
